# Protocol for project MIME: Motivation, inflammation, and Mood in Emerging Adults

**DOI:** 10.1016/j.bbih.2022.100520

**Published:** 2022-09-24

**Authors:** Daniel P. Moriarity, Marin M. Kautz, Kubarah Ghias, Kirsta Pennypacker, Eddie Harmon-Jones, Lauren B. Alloy

**Affiliations:** a Department of Psychiatry and Biobehavioral Sciences, University of California, Los Angeles, United States; b Department of Genetics, Stanford University, United States; c Department of Psychology and Neuroscience, Temple University, United States; d Department of Psychology, The University of New South Wales, Sydney, United States

**Keywords:** Depression, Bipolar disorder, Hypomania, Mania, Inflammation, Rumination, Cognitive vulnerabilities, Reward sensitivity, Anger incentive delay task, Stress

## Abstract

**Background:**

Atypical inflammatory biology is gaining evidence as a risk factor for mood psychopathology; however, little work has attempted to integrate inflammation into extant psychosocial frameworks of risk. Recent work using secondary data analysis has investigated the possibility of an immunocognitive model of mood disorders, in which cognitive vulnerabilities (i.e., rumination on positive or negative affect) increase the effect that arousal-related characteristics (e.g., reward sensitivity) have on inflammatory biology in ways that may confer risk for depression and hypo/mania symptoms. Project MIME (Motivation, Inflammation, and Mood in Emerging Adults) was designed to test this model in the context of a novel, reward-salient stressor (the Anger Incentive Delay Task, AIDT).

**Methods:**

This NIMH-funded study will result in a dataset of approximately 100 college undergraduates from a large university in Pennsylvania, United States of America. Eligible participants are recruited from an online screener, have to be 18–22 years old, fluent in English, and successfully answer several items designed to test whether participants randomly answer questions on the screener. Eligible participants are invited to an in-person visit in which they completed the AIDT, blood draws pre- and 50 minutes post-AIDT, and self-report questionnaires. Participants also complete a set of online questionnaires two weeks after the in-person visit.

**Discussion:**

Consistent with calls from the NIH director, this study seeks to diversify the tools used in stress research by validating a novel reward-salient stressor (in contrast to the field's reliance on social stressors) with respect to affective and immunological stress reactivity. In addition to this methodological goal, Project MIME is the first study specifically designed to test the immunocognitive model of mood psychopathology. Given the integration of several malleable treatment targets (approach behavior, emotion regulation, inflammation) into this model, results from this study could inform comprehensive, flexible intervention strategies for mood disorder prevention and treatment.

## List of abbreviations:

Motivation, Inflammation, and Mood in Emerging Adults: (MIME)
Trier Social Stress Task: (TSST)
Behavioral Activation System: (BAS)
Interleukin: (IL)
Anger Incentive Delay Task: (AIDT)
Monetary Incentive Delay: (MID)

## 1 Background

Increasingly, inflammation is conceptualized as a biological mediator partially mediating the relationship between perceived stress and psychopathology (Miller et al., 2009; Slavich and Irwin, 2014). However, research investigating this idea is limited by a lack of diversity in acute stress paradigms, a significant enough weakness across fields that the National Institute of Health's Director has made an explicit call for “expanding the experimental systems used to study stress and its effects” (Simmons et al., 2021). Specifically, psychoneuroimmunology studies heavily feature variations of the Trier Social Stress Task (TSST; Kirschbaum et al., 1993), which involves participants preparing and delivering a public presentation. Given evidence that inflammation and reward sensitivity/processing are related (Chat et al., 2021; Felger et al., 2016; Moriarity et al., 2020a; Moriarity et al., 2020b), the development of a reward-salient stressor is an important step forward for immunopsychiatry and stress research at large. Specifically, a validated reward-salient stress task would facilitate research in which theories about reward sensitivity, processing, and/or goal frustration can be tested experimentally with a complementary stressor. The primary goals of Project MIME (Motivation, Inflammation, and Mood in Emerging Adults) are to a) validate a modified version of the Anger Incentive Delay Task (AIDT; Angus and Harmon-Jones, 2019) suitable for pre-/post-stressor designs and b) test reward-salient stress reactivity as a mechanism of mood symptom risk in the context of an integrated immunocognitive model of mood psychopathology (Moriarity et al., 2018).

### 1.1 Reward sensitivity, inflammation, and the mood symptom spectrum

Atypical reward sensitivity/processing has been associated with psychopathology at both ends of the mood spectrum (i.e., hypo/mania and depression; Alloy et al., 2012, 2016; Henriques and Davidson, 2000; Morgan et al., 2013). The behavioral approach system (BAS)/reward hypersensitivity theory of bipolar spectrum disorders (Alloy et al., 2015, 2016; Johnson et al., 2012) describes that reward hypersensitivity may increase risk for hypo/mania symptoms by triggering “excessive reward activation states” associated with goal-striving and attainment. In contrast, reward hypersensitivity confers risk for irritability and/or depression by exacerbating “excessive reward deactivation states” to goal failure/frustration (Carver, 2004; Harmon-Jones, 2003). Although there is a large body of research supporting direct relationships between reward hypersensitivity and hypo/mania symptoms (Alloy et al., 2008, 2012; Boland et al., 2016; Francis-Raniere et al., 2006), evidence is less consistent regarding whether reward hypersensitivity is associated with depression symptom vulnerability (Alloy et al., 2016; Nusslock and Alloy, 2017). As suggested by Boland et al. (2016), this could be explained by reward hypersensitivity operating as a more distal risk factor for depression, mediated by other mechanisms (e.g., social rhythm disruption).

Critically, the reward/BAS model of bipolar disorders describes arousal (both positive and negative) as a driving force connecting reward sensitivity and mood symptoms. Thus, it is plausible that stress-reactive physiology associated with both bipolar disorder and depression might function as a mediator of this reward—symptom pathway. Specifically, both bipolar spectrum disorders (Modabbernia et al., 2013) and unipolar depression (Dowlati et al., 2010) are associated with atypical levels of inflammatory proteins. In support of this theory, reward sensitivity is associated with both basal levels of inflammatory proteins and acute inflammatory activity (Boyle et al., 2020; Eisenberger et al., 2017). Further, reward hypersensitivity is associated with elevated affective reactivity to stressors (Carver, 2004; Harmon-Jones, 2003; Hundt et al., 2013), which itself is associated with greater inflammatory stress reactivity (Carroll et al., 2011). Thus, symptoms at either end of the mood spectrum might be an example of multifinality of a pathway going from reward sensitivity to arousal and through inflammation.

### 1.2 Immunocognitive model of psychopathology

Immunopsychiatry primarily is dominated by studies of main effects between inflammatory proteins and mental health, despite many eminent etiological theories conceptualizing inflammation as a mediator between psychosocial stress and psychopathology (e.g., Slavich, 2020; Slavich and Irwin, 2014). Consequently, there is a need for datasets designed to investigate stress-modulation models of inflammatory risk for psychopathology (Moriarity, 2021). Identifying malleable psychosocial mechanisms that might influence the physiological stress response could provide insight into how psychosocial interventions may influence inflammatory profiles (Shields et al., 2020) and what psychosocial characteristics might exacerbate inflammatory risk for psychopathology.

There is initial evidence that cognitive vulnerabilities (especially rumination) predict higher levels of inflammatory proteins and higher inflammatory stress reactivity (Szabo et al., 2022; Zoccola et al., 2014). Further, adaptive cognitive response styles (e.g., positive engagement coping), might buffer the relationship between perceived stress and inflammatory biology (Low et al., 2013). Similarly, other research has found that rumination can moderate the relationship between stress/arousal-related characteristics and both depression and hypo/manic mood symptoms (Cohen et al., 2014; Reilly-Harrington et al., 1999). This work has recently been expanded into a preliminary “immunocognitive model of psychopathology” in which cognitive vulnerabilities amplify the effect arousal-related traits (e.g., reward sensitivity, anxiety) have on inflammatory biology in ways that confer risk for psychopathology. Moriarity et al. (2018) found initial support for this model in a sample of adolescents. Specifically, higher anxiety symptoms predicted greater changes in interleukin (IL)-6, which mediated the relation between baseline anxiety and changes in depression symptoms, but only when rumination was included as a moderator (this pathway was stronger in adolescents who were more likely to ruminate on negative experiences).

Particularly relevant to this study, two other studies found support for reward × rumination interactions consistent with segments of the immunocognitive model. In the same sample of adolescents as Moriarity et al. (2018), Moriarity et al. (2020) found that high reward drive (the facet of reward sensitivity concerning pursuit of goals) interacted with high rumination on negative affect to predict greater increases in IL-6 in response to the TSST (a social stressor focusing on giving a quality presentation in front of an audience). Further, non-perseverative cognitive responses (i.e., problem solving and distraction) both buffered the relation between reward drive and IL-6 stress reactivity. In a separate sample of emerging adults, interactions between reward responsivity and perseverative response styles (on both negative and positive affect) predicted i) inflammatory proteins, ii) depression symptoms, and iii) hypo/mania symptoms (Moriarity et al., 2020).

### 1.3 The present study

In sum, the goals of Project MIME are both methodological and applied in nature. First, this will be the first study to validate a reward-salient stressor (the AIDT) modified to be suitable for pre-/post-stressor designs. Second, the ability of this stressor to induce both affective and inflammatory reactions will be evaluated. Third, it will be tested whether i) reward sensitivity, ii) rumination, and iii) their interaction predict individual differences in reward-salient stress reactivity. Fourth, both studies that tested interactions between reward sensitivity and rumination predicting inflammatory proteins and mood symptoms described above will be replicated in a study specifically designed to address the original studies’ limitations. Fifth, if statistical power permits, a full immunocognitive model will be tested using moderated mediation models. These models will feature baseline reward sensitivity as the focal predictor, affective and inflammatory stress reactivity as mediators (in separate models), perseverative cognitive styles as a moderator of the relation between reward sensitivity and stress reactivity, and mood symptoms as the outcome.

## 2 Method

### 2.1 Participants

Participants are undergraduate students at a large university in Pennsylvania, United States of America. Participants are recruited using a screener on a department-run website and must be aged 18–22 at the time of screening. To the extent possible given recruitment difficulties during the COVID-19 pandemic, participants will be oversampled for high and low reward sensitivity (defined as the top and bottom 33% of BAS total score, as quantified in the first 428 participants to complete the screener). To participate, participants must report fluency in English and be comfortable having their blood taken. Participants are excluded if they incorrectly answer more than one out of three attention checks (items which instruct the participant how to respond to ensure they are carefully reading items) during the screener.

### 2.2 Procedure

**Fig. 1:**
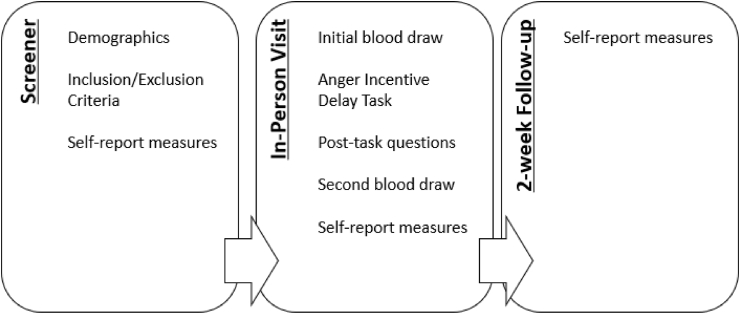
Study timeline.

Data collection involves an initial online screener on a department-run website, an in-person visit, and a 2-week follow-up (see Fig. 1).

#### 2.2.1 Screener

The screener includes an initial informed consent, describes the study, collects demographics, assesses inclusion/exclusion criteria, and included multiple self-report measures (see Table 1 for a timetable of self-report measures).

**Table 1:** Measure timetable.

Measures	Screener	In-Person Visit	2-week Follow-up
**Demographics**			
Birthdate	X		
Gender + Sex	X		
Race + Ethnicity	X		
Parental Income + SES	X		
Sexual Orientation	X		
**Medical Status**			
Current Medications	X	X	
Medical Conditions	X	X	
Psychiatric Treatment History	X		
Psychiatric Family History	X		
Height		X	
Weight		X	
Body Fat Percentage		X	
Time of Last Meal		X	
Waist-to-Hip Ratio		X	
Menstrual Status		X	
International Physical Activity Questionnaire (IPAQ)		X	
**Primary Self-Report Measures**			
Behavioral Inhibition/Behavioral Activation Scales (BIS/BAS)	X	X	X
Sensitivity to Punishment/Sensitivity to Reward Questionnaire (SPSRQ)	X	X	X
Ruminative Response Scale (RRS)	X	X	X
Responses to Positive Affect Scale (RPAS)	X	X	X
Patient Reported Outcomes Information System (PROMIS) Depression	X	X	X
Altman Self-Rating Mania Scale (ASRM)	X	X	X
AIDT validation items		X	
Self-Reported Affect		X	
Brief State Rumination Inventory (BSRI)		X	
**Secondary Self-Report Measures**			
Beck Suicide Scale (BSS)	X	X	X
PROMIS Anxiety	X	X	X
PROMIS Positive Affect	X	X	X
PROMIS Anger	X	X	X
Positive Valence Systems Scale (PVSS)	X	X	X
Cognitive Fusion Questionnaire (CFQ)	X	X	X
Traumatic Life Events Questionnaire (TLEQ)	X		
Childhood Trauma Questionnaire (CTQ)	X		
Acceptance and Action Questionnaire (AAQ)-II	X		X
Multidimensional Psychological Flexibility Inventory (MPFI)	X		X
Temporal Experience of Pleasure Scale (TEPS)	X	X	X
Cognitive Appraisal Scale (CAS)	X		
Emotional Reactivity Scale (ERS)	X	X	X
Self-Rating Scale (SRS)	X	X	X
Stress Appraisal Measure (SAM)	X		
Posttraumatic Growth Inventory-Short Form (PTGI-SF)	X		
Centrality of Event Scale (CES)	X		
PTSD Checklist for DSM-5 (PCL-5)	X		
Big 5 Inventory (BFI)		X	
Morningness/Eveningness Questionnaire (MEQ)		X	X
Adolescent Alcohol and Drug Involvement Scale (AADIS)		X	X
Difficulties in Emotion Regulation Scale (DERS)		X	X
Perceived Stress Scale (PSS)		X	X
General Behavior Inventory-10 Revised (GBI-10R)		X	X
The Behavior Inventory of Executive Function—Adult Version (BRIEF-A)		X	X
Acquired Capacity for Suicide Scale (ACSS)			X
Deliberate Self-Harm Inventory (DSHI)			X
Urgency, Premeditation, Perseverance, Sensation Seeking, Positive Urgency, Impulsive Behavior Scale (UPPS–P)			X

Note: Please note that not all measures were given to all participants. Some measures were added for portions of the study based off current research assistants' interests.

#### 2.2.2 In-person visit

Eligible participants are invited to an in-person study visit including a fasted pre-AIDT venipuncture blood draw, the AIDT, a post-AIDT blood draw 50 minutes after the stressor finished, and additional self-report questionnaires.

**Anger Incentive Delay Task (AIDT;**Angus and Harmon-Jones, 2019). The AIDT is a modified Monetary Incentive Delay Task (MID; Knutson et al., 2000) completed on a computer. Participants are instructed to “win as much money as possible and avoid losing money”, and that they can win money on some trials and avoid losing money on others by responding to a target stimulus (four white asterisks). Participants are told that if they finish the task with positive money, they will earn a $15 Amazon gift card. Participants start with $1 and can win or lose between $.20 and $.40 on each trial. At the beginning of each trial, a fixation cross is presented in the center of the screen for 900–1100 ms, followed by an incentive cue for 500 ms. Incentive cues are circles indicating that the participant can win money or triangles indicating that the participant can break even if the trial is successful (otherwise the participant will lose money). After the incentive cue, another fixation cross is presented for 1300–1700 ms. The target stimulus duration adapts to participants’ performance, increasing or decreasing by 20 ms to a minimum of 200 ms or a maximum of 340 ms if participant accuracy increases or decreases from 90%. Success probability will be manipulated further by increasing target stimulus duration by 40–80 ms on trials designed for success and decreasing by 40–80 ms on trials designed for failure. Targets remain on screen for the entire duration regardless of reaction time. After the target stimulus disappears, another fixation cross is presented for 450–550 ms, followed by a feedback stimulus presented for 1000 ms to indicate success or failure. Failures are signaled with a downward arrow; trials in which the participant wins money are signaled with an upward arrow; and trials in which the participant breaks even are signaled with an = sign. Following the feedback stimulus, another fixation cross is displayed for 450–550 ms, followed by the amount of money won or lost on that trial, displayed for 1000 ms.

During the final four blocks (out of twelve total), correct responses to trials with anticipatory stimuli indicating the possibility to win money begin to actually lose money on 58% of trials, despite presentation of successful feedback stimuli, to induce goal frustration. This change is not communicated to participants ahead of time. The entire task will consist of 228 trials across 12 blocks consisting of 19 trials each. The order of the first 18 trials in each block is pseudorandomized, and the same order will be presented to all participants. The final trial of each block is randomized to reduce the possibility of order effects. The AIDT can induce negative state affect and physiological responses (P3b amplitudes) to the goal-frustration blocks (Angus and Harmon-Jones, 2019). State affect and motivation to perform well is measured before the task and after each block of the task using 7-point scales on how intensely participants feel several emotions (e.g., sadness, anxiety, anger). Immediately following the task, participants complete the Brief State Rumination Inventory (Marchetti et al., 2018) and questions about their reactions to specific scenarios in the task (e.g., when the game informed them they answered correctly but still lost money).

**Blood Draws.** The first fasted blood draw is timed for immediately after completing informed consent at the start of the study. After the first blood draw, participants start the AIDT. Fifty minutes after the AIDT, participants provide a second blood sample. Blood samples are obtained via antecubital venipuncture by a certified phlebotomist into a 5 mL BD vacutainer Plasma Preparation Tube. Samples are spun at 1300 revolutions per minute for 10 minutes. Vacutainers are stored in an ultracold freezer at −80 °C and will be thawed on the day of assay. At each blood draw, the time of collection and participants’ relevant health metrics (e.g., BMI, body fat percentage) will be recorded.

#### 2.2.3 2-Week follow-up

Participants complete a follow-up survey 2-weeks after the study visit to assess change in psychosocial variables (see Table 1 for a timetable of self-report measures).

## 3 Discussion

The data collected in this study hold promise to advance two primary goals. First, this study was designed for the explicit purpose of testing theories integrating inflammatory physiology into established, robust risk pathways (i.e., rumination and reward sensitivity) for mood psychopathology. Ideally, theoretical integration of psychosocial and biological risk factors will allow for maximally comprehensive treatment plans, advancing precision medicine (Kautz, 2021; Moriarity, 2021). Three previously published studies from our team directly support parts of this model (Moriarity et al., 2018; Moriarity et al., 2020; Moriarity et al., 2020); however, they each had limitations inherent in secondary data analysis that will be addressed in this dataset. Second, in line with the NIH Director's calls for action (Simmons et al., 2021), this study aims to diversify the laboratory-based tasks available to stress researchers to include a reward-salient stressor suitable for pre-/post-stressor designs. We hope this will facilitate lasting impact even beyond the fields of immunopsychiatry and mood disorder research.

However, results from this study should be considered in light of several limitations. First, it would be ideal to be able to take multiple post-AIDT blood draws to evaluate peak reactivity windows. Second, having more than one follow-up would facilitate evaluation of how predictors are associated with longer-term, and potentially non-linear, trends in mood symptoms. Third, it would be ideal to be able to compare affective and inflammatory reactivity between the AIDT and the TSST (due to its status as the gold standard acute laboratory stressor) among the same individuals. Unfortunately, these opportunities are not feasible with the budget provided by an NRSA F31 training grant and thus, will be critical next steps for this line of work.

## Ethics approval and consent to participate

Ethics approval was granted by the Temple University IRB for this study. Written informed consent is collected for all participants.

## Consent for publication

Not applicable.

## Availability of data and materials

Data will be published as supplementary materials in subsequent publications and included as part of the peer review process. The corresponding author also can be contacted about the dataset as a whole.

## Funding

Daniel P. Moriarity was supported by National Research Service Award F31 MH122116 (the primary grant supporting Daniel Moriarity's salary and the execution of Project MIME), an APF Visionary Grant (which provided supplemental funds for Project MIME), and grant #OPR21101 from the California Initiative to Advance Precision Medicine (which has funded Daniel Moriarity's salary since starting his postdoctoral fellowship). Lauren B. Alloy was supported by National Institute of Mental Health R01 MH101168 and R01 MH123473 (both of which paid for lab infrastructure and study staff that supported Project MIME).

## Authors’ contributions

DPM generated the research questions and methodology for the study, wrote the primary grant funding its execution, managed participant recruitment and scheduling, ran participants, and recruited and trained research assistants. MMK secured additional funding for the execution of this study, ran participants, and recruited and trained research assistants. KG and KP both ran participants and were instrumental in maintaining a running project throughout the COVID-19 pandemic. EHJ assisted in the conceptualization and implementation of the modified AIDT for this study. LBA provided feedback on many drafts of the grant applications that funded this study, provided lab space and resources, and much mentorship and guidance throughout the execution of this grant.

## Declaration of competing interest

None.
